# Vagus nerve stimulation for spike-and-wave activation in sleep in a pediatric patient: a case report

**DOI:** 10.3389/fnins.2025.1675783

**Published:** 2025-10-23

**Authors:** Chunyan Zhao, Jiayi Ma, Han Xie, Yuying Pan, Yuwu Jiang, Lixin Cai, Qingzhu Liu, Ye Wu

**Affiliations:** ^1^Department of Pediatric Neurology, Children’s Medical Center, Peking University First Hospital, Beijing, China; ^2^Pediatric Epilepsy Center, Children’s Medical Center, Peking University First Hospital, Beijing, China

**Keywords:** vagus nerve stimulation, VNS, spike-and-wave activation in sleep, SWAS, developmental and epileptic encephalopathy/epileptic encephalopathy with spike-and-wave activation in sleep

## Abstract

**Background:**

Evidence regarding the efficacy of vagus nerve stimulation (VNS) in treating developmental and epileptic encephalopathy/ epileptic encephalopathy with spike-and-wave activation in sleep (DEE/EE-SWAS), particularly its impact on the SWAS remains limited. We present a boy with EE-SWAS who was treated with VNS at 4.8 years of age.

**Case presentation:**

A male patient developed seizures at 2.3 years of age. At 2.8 years of age, electroencephalography (EEG) showed SWAS, leading to regression in cognitive, motor, and language functions. Administration of multiple anti-seizure medications (ASMs) achieved poor efficacy, and repeated corticosteroids resulted in only transient improvement. He was treated with VNS at 4.8 years of age. Seizure freedom was achieved at 1.3 years postoperatively. The SWAS pattern was not observed on follow-up EEG 2 years after implantation. Concurrently, his neurodevelopment improved. No new ASMs or corticosteroids were added during this period. VNS was interrupted due to pulse generator battery depletion at 5.6 years after implantation. Increased SWI was showed on the EEG 6 months after the interruption of stimulation.

**Conclusion:**

Early VNS intervention should be considered in addition to conventional medication for young children with SWAS who have greater distance from the self-limited age.

## 1 Introduction

Spike-and-wave activation in sleep (SWAS) is a specific electroencephalographic (EEG) pattern characterized by near continuous spikes and waves induced by sleep (Specchio et al., 2022; Rubboli et al., 2023). This pattern was first described and termed electrical status epilepticus during sleep (ESES) by Patry et al. (1971). In the updated classification of epilepsy syndrome by the International League Against Epilepsy (ILAE), ESES was officially renamed SWAS. The initial diagnostic criterion proposed by Patry et al. (1971) required a spike–wave index (SWI) ≥ 85%. Emerging evidence suggested that cognitive or behavioral regression may also occur with lower SWI values. Based on this, de Boer et al. recommended a SWI ≥ 50% as the cutoff value for diagnosis of SWAS (Scheltens-de Boer, 2009). Although SWAS may spontaneously resolve around puberty, persistent SWAS can cause significant brain impairment, with the duration of SWAS showing a strong correlation with the degree of long-term neurocognitive deficits (Kramer et al., 2009; Peltola et al., 2011). Early therapeutic intervention to control SWAS is therefore crucial for long-term brain function outcomes. Current clinical management of SWAS faces dual challenges: conventional anti-seizure medications (ASMs) often demonstrate limited efficacy, while alternative therapies including corticosteroids and benzodiazepines, though showing moderate effectiveness, are associated with high relapse rates (41–89%) and significant adverse effects (Buzatu et al., 2009; Wiwattanadittakul et al., 2020).

Vagus nerve stimulation (VNS), a well-established neuromodulation therapy for drug-resistant epilepsy (DRE), demonstrates an overall response rate of 56.4% and a seizure-free rate of 11.6% in a meta-analysis involving 3,474 pediatric patients with DRE (Jain and Arya, 2021). In pediatric epilepsy syndromes such as Lennox–Gastaut syndrome (LGS) and Dravet syndrome, the response rates were 58.2 and 44.7%, respectively, (Jain and Arya, 2021). However, evidence of VNS efficacy remains limited for DEE/EE-SWAS, particularly its impact on SWAS (Park, 2003; Arhan et al., 2015; Carosella et al., 2016). We describe a boy with EE-SWAS who underwent VNS implantation at 4.8 years of age. He achieved seizure freedom, SWAS resolution and developmental progress. Through long-term follow-up, SWI increased was observed after stimulation interruption due to pulse generator battery depletion. Here, we use SWI ≥ 50% as the criterion for diagnosing SWAS.

## 2 Case presentation

A 11-year-old boy had an unremarkable birth history. At 5 months of age, he developed impaired consciousness and generalized tonic–clonic seizures during a febrile illness. Cerebrospinal fluid (CSF) analysis revealed a white blood cell count of 10 × 10⁶/L and a protein level of 0.46 g/L, leading to a diagnosis of viral encephalitis. His symptoms resolved within 2 days. He remained well until 2.3 years of age. At 2.3 years of age, he began experiencing monthly focal preserved consciousness seizures, characterized by either the left leg twitching or left-sided facial twitching. Subsequently, seizure frequency increased progressively. At 2.8 years of age, EEG revealed SWAS and atypical absence seizures. Concurrently, he manifested neurodevelopmental regression, including an inability to stand or walk independently, language regression, and cognitive decline. He was treated with multiple anti-seizure medications (ASMs), including levetiracetam, sodium valproate, lamotrigine, topiramate, clonazepam, sultiame, and clobazam, but with poor efficacy. Three courses of pulsed corticosteroids at 2.9, 3.3, and 4 years of age produced transient efficacy. However, all gains were followed by relapse.

**Figure 1:**
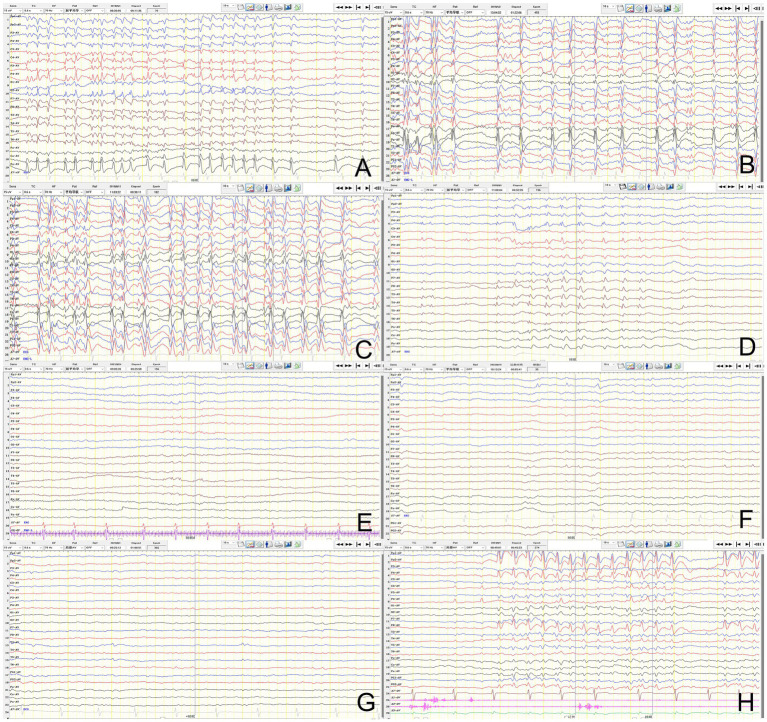
Evolution of spike-and-wave activation in sleep (SWAS) after VNS implantation. **(A)** Baseline sleep EEG before VNS implantation showing SWAS pattern. **(B,C)** SWAS remained on follow-up sleep EEG at 0.5 and 1 year after VNS. **(D–G)** SWAS resolved on follow-up sleep EEG at 2, 3, 4, and 5 years after VNS. **(H)** Increased spike-wave index was showed on the EEG 6 months after the interruption of stimulation.

Preoperative VNS assessment revealed that the patient exhibited approximately 30 focal motor seizures monthly, inability to stand or walk independently, markedly reduced speech output, and impaired cognition. Brain MRI showed bilateral atrophy of the putamen and caudate nucleus. Ambulatory 4-h EEG revealed spike-and-wave complexes in the centroparietal midline and Rolandic regions, with a SWI of 80% (Figure 1A). Griffiths mental development scales confirmed severe global developmental delay (DQ/IQ ≤ 35). Concurrent ASMs included valproate, clobazam, and levetiracetam.

VNS implantation was performed successfully at 4.8 years of age. The vagus nerve stimulator was turned on 14 days after implantation. VNS was initially started at: output current 0.5 mA, pulse width 500 μs, frequency 30 Hz, on time 30 s, and off time 5 min. At 6 months postoperatively, the output current was increased to 1.5 mA, with the remaining parameters unchanged. The patient’s seizure frequency decreased to 15 times monthly (a 50% reduction), with SWAS (SWI 80%) persisting on the repeated ambulatory 4-h EEG (Figure 1B). At 1 year postoperatively, the output current was further titrated to 2.0 mA. His seizure frequency decreased to 10 times monthly, with SWAS (SWI 75%) remained (Figure 1C). At 2 years postoperatively, the parameters were optimized to: output current 1.8 mA, pulse width 500 μs, frequency 30 Hz, on time 30 s, and off time 3 min. He had maintained seizure freedom for 7 months, with resolution of SWAS on repeat EEG (Figure 1D). The SWI decreased to 20%. No new ASMs or corticosteroids were introduced during VNS. The parameters remained stable thereafter. He maintained seizure freedom, with decreased spike-and-wave discharges on subsequent EEG (Figures 1E–G).

**Figure 2:**
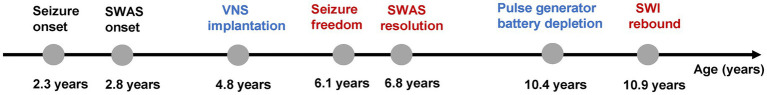
Timeline of the patient’s clinical course.

At 5.6 years postoperatively, the pulse generator battery was depleted. The patient remained seizure freedom. However, the SWI had increased to 30% on the ambulatory 4-h EEG 6 months after the interruption of stimulation (Figure 1H). Given his current age of 11 years, we decided to continue close follow-up without replacing the battery. The timeline of key events in the patient’s clinical course is presented in Figure 2.

## 3 Discussion

SWAS is an EEG phenomenon characterized by nearly continuous spikes and waves during non-rapid eye movement (NREM) sleep, typically observed in pediatric epilepsy syndromes associated with regression of cognitive, behavioral, linguistic, and motor functions. In 2022, ILAE reclassified this syndrome as DEE/EE-SWAS, including previously recognized entities such as epilepsy with continuous spike–wave during slow-wave sleep (CSWS), variants of self-limited epilepsy with centrotemporal spikes (SeLECTS), and Landau–Kleffner syndrome (LKS) (Specchio et al., 2022). DEE/EE-SWAS accounts for approximately 0.2–1% of all pediatric epilepsy cases (Nickels and Wirrell, 2008). The underlying mechanisms of SWAS remain incompletely elucidated. SWAS has been associated with diverse etiologies, including structural, genetic, and immune-mediated epilepsies. Structural etiologies predominate, encompassing thalamic lesions and malformations of cortical development such as polymicrogyria (Andrade-Machado, 2012; Sonnek et al., 2021). Genetic etiologies involve pathogenic variants in *GRIN2A*, *SCN2A*, *KCNA2*, *KCNQ2*, *SLC6A1*, *SLC9A6*, and copy number variations linked to 15q13.3 and 16p11.2 microdeletion syndromes (Dimassi et al., 2014; Gong et al., 2021). The therapeutic response to corticosteroids further suggests potential involvement of inflammatory mechanisms in the pathogenesis.

The duration of SWAS correlates with cognitive impairment (Seegmüller et al., 2012; Pera et al., 2013). There is no universally recommended treatment regimen. ASMs are generally ineffective. Although benzodiazepines and corticosteroids exhibit partial clinical responsiveness, their utility is constrained by significant adverse effects and high SWAS relapse rates. Surgery may resolve SWAS, but is effective only in children who have failed medical therapy and have a resectable structural etiology. However, surgery carries the risk of causing hemiplegia, hemianopia, and permanent hand dysfunction (van den Munckhof et al., 2015; Sonnek et al., 2021; Wu et al., 2024). To optimize cognitive preservation, early alternative therapeutic strategies are strongly recommended for younger children with persisting clinical seizures and SWAS despite adequate ASMs and corticosteroids therapy.

The therapeutic role of VNS in DEE/EE-SWAS remains inadequately defined, especially on SWAS pattern, with only few cases reported to date. Carosella et al. (2016) reported a 12 years EE-SWAS girl with structural etiology achieving seizure freedom at 5 months and SWAS resolution at 8 months post-VNS. Given that she was close to the self-limited age, it was difficult to distinguish whether the improvement was attributed to the efficacy of VNS or the natural course. In a cohort of 6 children with LKS treated with VNS, the mean implantation age was 10.3 ± 4.2 years (range 6–16 years). Park (2003) observed that 2 children experienced a reduction ≥ 50% in seizure frequency at 3 months post-VNS, 3 children experienced a reduction ≥ 50% in seizure frequency at 6 months post-VNS, though SWAS outcomes were unreported. Arhan et al. (2015) reported two children with DEE/EE-SWAS treated with VNS, one achieved a > 75% reduction in seizure frequency at 4 months after VNS implantation, maintained to the final follow-up at 3 years post-VNS. And the other patient achieved a 50% reduction in seizure frequency at 6 months post-VNS, but seizure frequency increased during follow-up. However, the age of implantation and the SWAS outcomes were not specified (Arhan et al., 2015).

DEE/EE-SWAS is age-dependent and usually self-limited around puberty. Seizure control is typically achieved after 12 years of disease course, while SWAS persists until a mean age of 11.1 years (Tassinari et al., 2000). In this case, the patient underwent VNS implantation at 4.8 years of age. The seizure frequency decreased rapidly and eventually achieved seizure freedom after VNS. The SWAS pattern resolved on repeated EEG at 2 years after VNS, accompanied by developmental progress. The age of SWAS resolution was earlier than the self-limited age. No new ASMs or corticosteroids were introduced during VNS. Therefore, the therapeutic effects may be reasonably attributed to VNS. Notably, we also observed a rebound in SWI after termination of stimulation due to generator battery depletion. This finding strengthens the evidence for VNS effects on SWAS.

The pathogenesis of SWAS is unclear. Some studies suggest that it is associated with thalamocortical system hyperactivation (Japaridze et al., 2016), while others implicate an imbalance between inhibitory neurons in the thalamic reticular nucleus and glutamatergic excitatory neurons in the dorsal thalamus (Beenhakker and Huguenard, 2009; Sánchez Fernández et al., 2012). Previous studies on the mechanism of VNS have also involved these pathways (Ricardo and Koh, 1978; Marrosu et al., 2003). However, the specific mechanism by which VNS directly effects on SWAS remains unclear.

In conclusion, the efficacy to VNS observed on the pediatric patient supports the consideration of early VNS intervention in addition to conventional medication for young children who have greater distance from the self-limited age. Future studies with larger sample sizes are warranted to confirm these findings.

## Data Availability

The original contributions presented in the study are included in the article/supplementary material, further inquiries can be directed to the corresponding author.
